# Comparison of Neurologic and Radiographic Outcomes with Solitaire versus Merci/Penumbra Systems for Acute Stroke Intervention

**DOI:** 10.1155/2013/715170

**Published:** 2013-12-30

**Authors:** Shannon Hann, Nohra Chalouhi, Robert Starke, Ashish Gandhe, Michael Koltz, Thana Theofanis, Pascal Jabbour, L. Fernando Gonzalez, Robert Rosenwasser, Stavropoula Tjoumakaris

**Affiliations:** ^1^Department of Neurosurgery, Thomas Jefferson University and Jefferson Hospital for Neuroscience, Philadelphia, PA 19107, USA; ^2^Department of Radiology, Thomas Jefferson University and Jefferson Hospital for Neuroscience, Philadelphia, PA 19107, USA

## Abstract

*Background and Purpose*. The Solitaire Flow Restoration was approved by the FDA in 2012 for mechanical thrombolysis of proximal occlusion of intracranial arteries. To compare the Solitaire FR device and the Merci/Penumbra (previously FDA approved) systems in terms of safety, clinical outcomes, and efficacy including radiographic brain parenchymal salvage. *Methods*. Thirty-one consecutive patients treated with the Solitaire and 20 patients with comparable baseline characteristics treated with Merci or Penumbra systems were included in the study. Primary outcome measures included recanalization rate and modified Rankin Scale score at followup. Secondary outcomes included length of procedure, incidence of symptomatic intracranial hemorrhage, 90-day mortality, and radiographic analysis of percentage area salvage. *Results*. Compared with the Merci/Penumbra group, the Solitaire group showed a statistically significant improvement in favorable outcomes (mRS ≤ 2) (69% versus 35%, *P* = 0.03) and symptomatic ICH rate (0 versus 15%, *P* = 0.05) with a trend towards higher recanalization rates (93.5% versus 75%, *P* = 0.096) and shorter length of procedure (58.5 min versus 70.8 min, *P* = 0.08). Radiographic comparison also showed a significantly larger area of salvage in the Solitaire group (81.9% versus 71.9%, *P* = 0.05). *Conclusion*. Our study suggests that the Solitaire system allows faster, safer, and more efficient thrombectomy than Merci or Penumbra systems.

## 1. Introduction

The goal of acute ischemic stroke treatment is arterial recanalization and restoration of brain parenchymal perfusion. Since the Food and Drug Administration (FDA) approval of tissue plasminogen activator (tPA) for treatment of acute ischemic stroke in 1996, intravenous tPA (ivtPA) administration within a 3–4.5 hours window postsroke has been the mainstream of stroke intervention [1].

However, ivtPA therapy has shortcomings including a limited administration window and less than ideal reperfusion outcome especially in large vessel occlusions. In fact, the recanalization rate with ivtPA is as low as 10% in internal carotid artery (ICA) occlusions and less than 30% for proximal middle cerebral artery (MCA) occlusions [2]. As such, intra-arterial thrombolysis within 6 hours and mechanical thrombectomy within 8 hours from symptom onset have been increasingly used to achieve faster and more efficient recanalizations of large arterial occlusions [1].

In the past decade, several randomized controlled trials for intra-arterial thrombolytic therapy and mechanical thrombolysis have been conducted and shown promising results. These trials led to the FDA approval of the first thrombectomy device, the Merci Retriever (Merci, Concentric Medical, Mountain View, CA) in 2004, and the second device, the Penumbra (Penumbra, Alameda, CA), in 2008. Although both devices are associated with relatively high rates of successful recanalization, large clots in major intracranial arteries remain quite resistant and require a prolonged time for recanalization [3]. The Solitaire Flow Restoration (FR) (ev3/Covidien, Irvine, CA) is a self-expanding, fully retrievable stent that obtained FDA approval in March 2012 based on the results of the Solitaire with the Intention for Thrombectomy (SWIFT) trial. This study found higher recanalization rates (61% versus 24%) and better neurologic outcomes (58% versus 33%) in the Solitaire FR device compared with the Merci device [4].

We present the first study comparing the Solitaire FR device and the Merci and/or Penumbra systems in terms of efficacy, safety, clinical outcomes, and area of territory at risk saved with revascularization by analyzing pre and postintervention imaging studies. We also reviewed published large scale mechanical thrombectomy trials for Merci, Penumbra, Merci 2, Solitaire, and TREVO devices to compare our Solitaire FR results with these various current devices.

## 2. Patients and Methods

### 2.1. Eligibility

**Figure 1:**
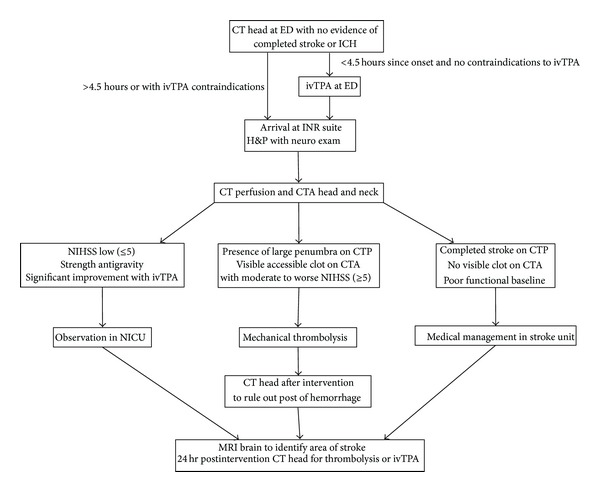
Thrombolysis protocol for acute ischemic stroke.

This is a single-center study of 31 patients treated with mechanical thrombectomy with the Solitaire FR device at the Jefferson Hospital for Neuroscience (JHN) in between March 2012 and November 2012. After neurological examination, suspected acute stroke patients are either admitted from Thomas Jefferson University Hospital emergency department, transferred from affiliated community hospitals, or directly accepted by the attending physician on stroke telemedicine call. Upon arrival to the JHN, patients are directly brought to the interventional neuroradiology (INR) suite for full history and physical exam by neurosurgical residents, fellows, and attending physicians. Subsequently, patients meeting clinical criteria for intervention with no CT evidence of completed stroke or hemorrhage proceed to immediate CT perfusion of head and CT angiogram of head and neck to determine the extent of territory at risk and to identify a major intracranial arterial occlusion. Based on the clinical signs, National Institute of Health Stroke Scale (NIHSS), and imaging findings the decision is made whether to perform mechanical thrombolysis or not.  Figure 1 details our patient selection protocol for thrombolysis. Major criteria for intervention include poor NIHSS ≥ 5, large territorial mismatch between cerebral blood volume and blood flow/mean transit time on CT perfusion scan, visible main arterial thrombus on CT angiogram, and worsening neurological performance since time of referral [5]. Our institution uses CT perfusion guided recanalization selection because it has reported lower intracranial hemorrhage rate and mortality rates compared with time guided selection [6]. Contraindications to interventions are improving neurological status, low NIHSS, and multiple medical comorbidities with poor functional baseline.

### 2.2. Procedures

Procedures are performed under general anesthesia and neuromonitoring with both somatosensory and motor evoke potentials. Access is obtained on the side contralateral to the intraluminal thrombus. The femoral artery is the first choice followed by the radial, brachial, and carotid arteries. The specific system used to support the Solitaire FR device may be variable depending on the patients peripheral vascular anatomy, presence of concurrent carotid pathology, clot location, and/or operator discretion. The majority of cases presented in this series achieved successful results with a 7F sheath and selective catheterization using a 0.038 Guide wire (Terumo, Somerset, NJ) and 6F Envoy guide catheter (Codman Neurovascular, Raynham, MA). Superselective catheterization of the target vessel was achieved with a 0.014 inch Synchro-2 microwire (Stryker; Fremont, CA) and Prowler Select Plus microcatheter (Cordis Neurovascular, Miami Lakes, FL).

Placement of the microcatheter distal to the thrombus is confirmed by microinjection of 60% contrast under digital subtraction angiography (DSA). The Solitaire FR device is then brought into the field and advanced through the microcatheter with biplane fluoroscopy to confirm its central position over the thrombus. The microcatheter is then pulled back to unsheathe the Solitaire FR device while maintaining a constant position of the Solitaire delivery wire. The Solitaire FR device is left completely unsheathed for 3–5 minutes. Once the appropriate time has elapsed, the proximal 1/4 of the Solitaire stent is retrieved within the microcatheter and then pulled out thru the guide catheter under continuous negative aspiration with a 50 mL syringe. Some operators prefer the 8F Merci Balloon Guide Catheter (Concentric Medical) so that it may be inflated before aspiration with the 50 mL syringe to aid in thrombus retrieval. The substitution of this guide catheter necessitates placement of an 8F sheath for access. Control angiograms are performed after Solitaire retrieval to confirm revascularization.

### 2.3. Outcome Measure

On admission, NIHSS and ivtPA administration status were checked; NIHSS was reassessed 24 hours after intervention and at time of discharge. Baseline modified Rankin Score (mRS) was obtained from family on admission and reassessed at ≥90 days during a follow-up office visit. CT head was performed within 24 h after intervention to diagnose hemorrhagic complications. MRI brain was also performed 24 h after intervention to document the area of completed stroke.

Primary outcome measures included recanalization rate and modified Rank in Scale score at followup. Secondary outcomes included length of procedure, incidence of symptomatic intracranial hemorrhage, 90-day mortality rate, and radiographic analysis of percentage area salvage. Successful recanalization was defined as a Thrombolysis In Myocardial Ischemia (TIMI) reperfusion grade of 2 or 3 on immediate postprocedural angiograms. The area of brain parenchymal salvage was documented by volumetric analysis. Specifically, the volume of completed stroke on MRI DWI sequence was subtracted from the volume of the territory at risk on initial CT perfusion. The volume of territory at risk was determined on initial CT head perfusion by measuring the mismatched area between mean transit time and blood volume in the axial plane and multiplying the total calculated area by the slice thickness of the corresponding image. The volume of completed stroke was measured on DWI signal abnormality from 24 hr postintervention MRI axial plane multiplied by the respective slice thickness with corresponding signal changes. The difference in the territory at risk on CT head perfusion study and DWI sequence in MRI brain is the area salvage. The percentage salvage is calculated using area salvage divided by the initial territory at risk. All calculations were carried out by a neuroradiologist with no prior knowledge of postoperative outcomes.  Figure 3 shows the typical imaging studies from a Solitaire patient. Safety outcome was assessed by (1) symptomatic intracranial hemorrhage after intervention, (2) device-induced damage to vessels and further propagation of thrombus, and (3) mortality rate at 90 days. Postprocedural groin hematoma was not included in safety outcome as this is a complication expected in any angiographic procedure; however, we did collect this data as procedure related complications.

The results from the Solitaire group were compared with those of the Merci/Penumbra group. Comparison group consisted of 20 patients treated with Merci and/or Penumbra system as first choice device from February 2010 to January 2011 at our institution. The characteristics of patients in the Merci, Penumbra group were comparable with the Solitaire group in terms of age, sex, medical comorbidities, and NIHSS in order to eliminate confounding variables. The focus of the comparison was not only the clinical outcomes but also the radiographic outcome of percentage area salvage; thus, patients with nondiagnostic CT perfusion study or with contraindications to MRI study such as those with a cardiac pacer were not included in the comparison group. After adjusting the confounding variables and availability of CT perfusion and MRI study, only twenty patients qualified to be included in the comparison group among the 44 total patients treated with Merci or penumbra system previously. Merci/Penumbra patients are grouped together because there were not enough patients treated in either group alone that would comprise a large enough comparison group.

### 2.4. Statistical Analysis

Data are presented as mean and range for continuous variables and as frequency for categorical variables. Analysis was carried out using unpaired *t*-test, Chi-square, and Fisher's exact tests as appropriate. Univariate analysis was used to test covariates predictive of the following dependent outcomes: unfavorable outcome (mRS 3–6) and TIMI-2 or 3. *P* values of ≤0.05 were considered statistically significant. Statistical analysis was carried out with Stata 10.0 (College Station, TX).

## 3. Results

### 3.1. Baseline Characteristics

Thirty-one consecutive patients with acute ischemic stroke treated with Solitaire FR device as a first-choice modality of mechanical thrombectomy were included. The mean patient age was 64.1 (range: 32–87) years and 23 (74%) were males. The NIHSS ranged from 3 to 26 (average 14, SD ± 6.02) on arrival. Arterial occlusion sites were as follows: M1 (51%), M2 (29%), ICA or T-occlusion (13%), and posterior circulation (6%).

Thirteen patients (42%) were treated with IV tPA prior to arrival; however, due to persistence of symptoms and identification of retrievable clot associated with a large area of territory at risk on CTA and CTP, Solitaire thrombectomy was performed.

### 3.2. Recanalization Rate

Table 1 summarizes the outcome of each patient along with the recanalization grade. Twenty-four patients (77%) were treated with the Solitaire FR as a sole thrombectomy device and seven patients underwent additional mode of treatment (i.e., Merci, Penumbra, Trevo, and/or intra-arterial TPA) to achieve maximum restoration of flow (TIMI 2 or 3). The mean time from stroke to recanalization was 8.03 ± 3.37 (SD) hours (range: 4–17 hours). The mean procedure time defined as time between arterial puncture to flow restoration observed on digital subtraction angiography (DSA) was 58.5 ± 22.9 (SD) minutes overall and only 40.3 ± 19.7 (SD) minutes for the successful procedures that did not require other mechanical thrombectomy rescue treatments. We obtained an overall recanalization rate (TIMI scores of 2 and 3) of 93.5%; treatment with the Solitaire FR device alone achieved a 79.3% recanalization rate. Only two patients failed to recanalize. One (patient 13) had a PCA thrombus subsequent to left vertebral artery dissection, the tortuous anatomy precluded passage of Solitaire FR device as well as proper deployment of stents across the dissection. The other (patient 15) had a high clot burden with a right ICA T-occlusion associated with distal M1 and M2 clots; despite 3 passes with the Solitaire FR device followed by thrombectomy with the Merci device and intra-arterial TPA injection, reperfusion could not be achieved at level of both the superior and inferior M2 branches. The number of device passes ranged from 1 to 5 (average 1.8, SD ± 1.4). There were no device fractures or arterial dissections. The NIHSS scores at 24 hours after intervention ranged from 0 to 26 (average 9.8, SD ± 6.9), while NIHSS at discharge ranged from 0 to 27 (average 7.2, SD ± 7.1).

### 3.3. Recanalization Grade and Improvements in NIHSS

At discharge, eighteen patients (58%) had a good outcome (NIHSS improvement of ≥5), six (19%) had a fair outcome (NIHSS improvement of 1–4), and seven (23%) had a poor outcome (no improvement or worsening NIHSS).  Table 2 summarizes patient recovery with respect to TIMI grade. Of note, 15 of 18 patients (83%) with good NIHSS outcome at discharge had TIMI 3, while 7 out of 13 patients (54%) with fair or poor outcome did not achieve complete recanalization. When this data was analyzed with Fisher's exact test, it was found that TIMI 3 perfusion was a statistically significant predictor of good outcome at discharge (*P* = 0.012).

### 3.4. Clinical Outcomes and Safety

**Figure 2:**
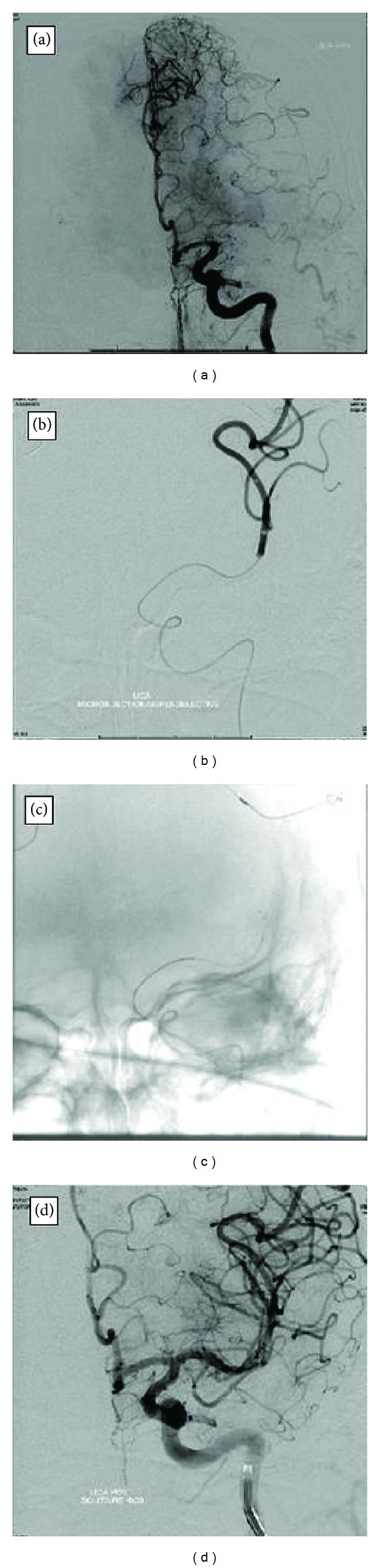
Case 14. (a) DSA of left ICA arterial phase. AP view shows an occlusion of the left M1. (b) Guiding catheter in left MCA, the distal tip of the microcatheter, and the microwire have crossed the occluded portion of the L M1 and moved through the thrombi into M2. This injection shows the distal end of the thrombus. (c) This AP view shows the terminal radiopaque marker of Solitaire FR device which indicates the start of deployment. (d) DSA of L ICA after the stent retrieval shows that the distal L MCA branches have been completely opened.

**Figure 3:**
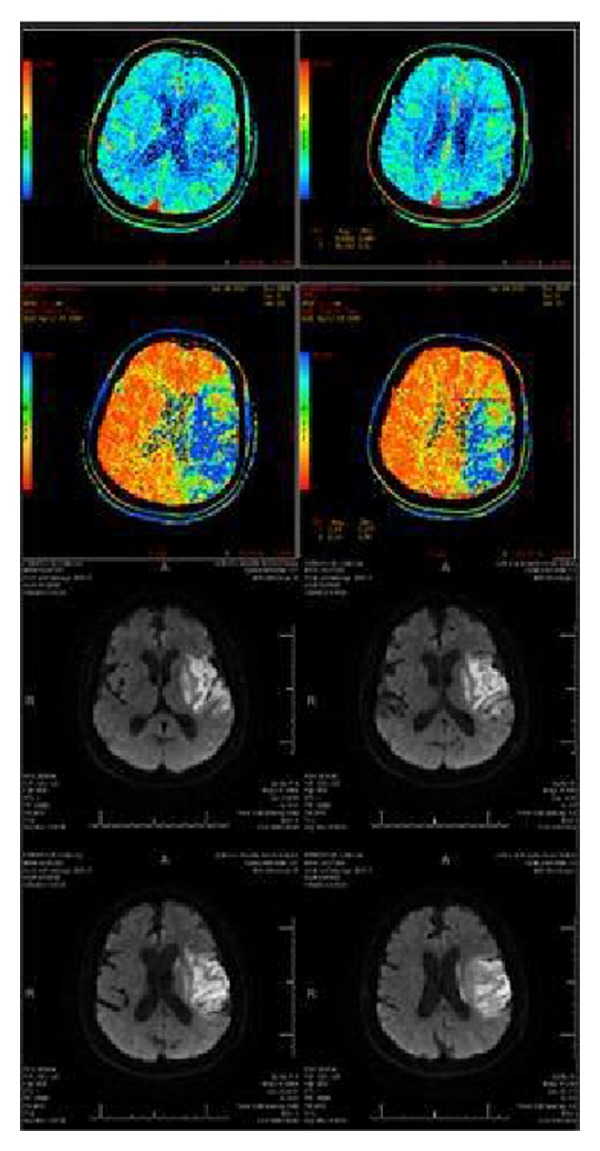
Top shows CT head perfusion study with mismatch in mean transit time and blood volume. Bottom shows smaller final completed stroke area in DWI sequence of MRI. Both used to calculate percentage area salvage.

At three to six months, 69% of the patients had mRS ≤ 2 (see Table 1). Two patients were lost to followup and were not included in the analysis. There was no treatment-related mortality. The overall mortality rate after 3–6 months of followup was 10.3% (total of three patients including one inpatient mortality due to malignant cerebral edema and family withdrew care). No symptomatic hemorrhagic complications were observed after intervention. Seven patients had asymptomatic petechial hemorrhage in the stroke territory on 24 hour postintervention CT head. Luxury perfusion (contrast medium enhancement in CT head) was observed in 13 cases (41.9%).  Figure 2 illustrates the typical Solitaire thrombectomy procedure in patient 14. Postprocedural minor groin hematoma occurred in 6 (Solitaire) versus 4 (Merci/Penumbra). Each group had three CAT scan documented retroperitoneal hematoma that did not require vascular surgical intervention.

### 3.5. Comparison with the Merci-Penumbra Group

A summary of patient characteristics stratified for both groups are presented in Table 3. These confounding factors were similar in both groups of patients. The radiographic recanalization rate and area salvage results of the Solitaire group are tabulated in Table 4 and those of the Merci/Penumbra group in Table 5.

#### 3.5.1. Recanalization Rate Comparison

In the Merci/Penumbra group, ten patients (50%) achieved a TIMI grade of 3, five patients (25%) achieved a TIMI grade of 2, and 5 failed to recanalize (3 TIMI1 and 2 TIMI0). Similarly to the Solitaire group, when the recanalization was not attained, rescue procedure was taken with another device or ia-TPA. Four patients required additional rescue treatment in this group. Average procedure time was 70.8 minutes.

There was a trend towards higher recanalization rates (TIMI 2-3) (93.5% versus 75%, *P* = 0.096) and shorter length of procedure (58.5 min versus 70.8 min, *P* = 0.08) in the Solitaire group versus the Merci/Penumbra group. However, no statistical difference was reached with respect to recanalization grade and time of procedure.

#### 3.5.2. Clinical Outcome Comparison

In the Merci/Penumbra group, the average 90-day mRS was 4 (SD ± 2). Seven of 20 (35.0%) patients had mRS ≤ 2. Three patients had symptomatic intracranial hemorrhage and the 90-day mortality rate was 45% (9 out of 20).

The 90-day mRS was significantly better in the Solitaire group with 69% versus 35% achieving mRS ≤ 2 (*P* = 0.03) and the hemorrhagic complication rate was significantly higher in the Merci/Penumbra group (0% versus 15%, *P* = 0.05).

#### 3.5.3. Area Salvage Comparison

The mean percentage salvage area was significantly larger in the Solitaire group (81.9% ± 17.6) versus the Merci/penumbra group (71.9% ± 19.7) (*P* = 0.05).

## 4. Discussion

### 4.1. Key Results and Interpretation

We assessed the safety, efficacy, and clinical outcome of the Solitaire FR system in our institution and compared these results with a group of patients treated with the Merci/Penumbra system. Most recently, two randomized clinical trials (SWIFT and TREVO2 trails) have compared newer clot retrieval devices in treatment of acute ischemic stroke. In the SWIFT study, patients were significantly more likely to have flow restoration (TIMI scale 2 or 3) and a favorable outcome with the Solitaire FR device compared with the Merci device [4]. In the TREVO2 trial, patients treated with the Trevo device were 4.2 times more likely to achieve revascularization, with a significantly higher rate of favorable outcomes compared with the Merci device [7]. In our study, we not only reproduced higher rates of flow restoration and favorable outcomes with the Solitaire device but also demonstrated that Solitaire patients attained radiographically a significantly higher percentage salvage rate than Merci/Penumbra patients.

Comparing the outcomes of the present study with the results of five large mechanical thrombolysis trials (see Table 6): Merci, Multi-Merci, Penumbra Pivotal, SWIFT, and TREVO2 trials, our study obtained a higher recanalization rate than the Merci, Multi-Merci, and Penumbra pivotal stroke trials [3, 4, 8]. Our recanalization results were similar to those of the SWIFT and TREVO2 trials. The clinical outcome as represented by mRS ≤ 2 and three-month mortality rates in our study were significantly improved compared with all of these studies.

We believe that radiographic analysis enables more direct comparison of treatment efficacy in restoring viable brain tissue without introducing confounding variables such as patients' age, baseline heath status, and recovery process during rehabilitation. Clinical outcome assessment by means of mRS tends to involve these confounding factors that modify recovery from stroke in addition to the results of thrombectomy. Thus, in a sense, area salvage analysis is a simpler measure for the efficacy of thrombectomy device. Of course, in clinical medicine, our ultimate goal is to provide better clinical outcome for patients and this necessitates correlation between area salvage and clinical recovery from stroke.

Other key findings include a longer procedure time and a higher incidence of symptomatic intracranial hemorrhage after intervention for Merci/Penumbra group. Taken together, these findings suggest that the Solitaire FR device allows safer, faster, and more efficient treatment of acute ischemic stroke patients than older devices used in our institute.

### 4.2. Limitations

Our study is limited by the small number of patients treated with old generation systems: Merci and Penumbra (only twenty patients). Because of this, we decided to combine patients treated with Merci and Penumbra systems together as a comparison group. Though this demonstrated that the newest system (Solitaire FR) is superior to the older systems, it does not effectively convey whether the Solitaire is better than the Merci or the Penumbra individually. This grouping does not mean that authors believe that the Merci and the Penumbra systems yield similar recanalization results, clinical, or radiographic outcomes, rather it is just a grouping constructed based on the time course of thrombolysis device used in our institution. Another limitation is baseline characteristics of the Solitaire group and the Merci/Penumbra groups are not matched due to the retrospective nature of the study and the limited number of patients. Furthermore, despite that authors do acknowledge the TREVO trial and its efficacy being reported to be comparable if not superior to the Solitaire, our institution does not currently have enough patients treated with TREVO system so they were not included in this study. Another inherent limitation of our study is its retrospective design. The main methodological limitation to our study relates to its small sample size causing the study to be underpowered to detect small differences in outcome. Our results reflect the experience of a single institution with specific protocols for mechanical thrombectomy and may not be entirely applicable to other centers.

## 5. Conclusion

We presented the first single-center study comparing the Solitaire FR device and the Merci/Penumbra systems. We found significantly higher recanalization rates and improved outcomes with a statistically significant increase in the percentage area salvaged among patients treated with the Solitaire device. Our study adds to the growing body of evidence supporting the safety and efficacy of mechanical thrombectomy with the Solitaire FR system in large proximal arterial occlusions.

## Figures and Tables

**Table 1:** Solitaire patient characteristics and treatment result.

Patient	Age	Gender	NIHSS on arrival	NIHSS at discharge	ivTPA	Location of thrombus	Time to reperfusion (hrs)	Procedure time (min)	Rescue treatment*	No. of pass	TIMI	F/U mRS
1	87	F	18	13	N	R M2	17	42	N	1	3	4
2	64	M	12	2	N	L M1	11	90	N	2	3	1
3	73	M	12	5	Y	L M1	7	36	N	1	3	2
4	32	M	18	0	Y	R M2	5	33	N	1	3	0
5	50	M	21	2	N	R ICA	5	65	N	1	2	1
6	52	M	7	1	N	Basilar	6	73	N	1	3	0
7	76	M	12	4	N	R M1	10	44	N	2	3	2
8	77	M	13	0	Y	R M1	6	65	N	2	2	0
9	62	M	9	5	N	L M2	5	70	N	1	2	2
10	77	F	13	5	Y	L ICA	10	80	Y (Penum/Plasty)	1	3	2
11	48	M	12	0	N	R M2	6	20	N	1	3	0
12	82	M	9	4	Y	L M1	5	26	N	1	3	4
13	69	M	9	9	N	L vert	6	65	N	1	1	4
14	63	F	21	13	N	L M1	5	41	N	2	3	1
15	87	M	19	27	Y	R ICA	5	54	Y (Merci, PLASTY)	4	0	6
16	63	M	18	12	N	R M1	11	52	N	1	3	4
17	71	M	13	1	Y	R ICA	7.5	57	Y (iaTPA)	1	2	0
18	74	M	10	Expired	N	R M1	16	105	N	2	3	6
19	61	M	15	12	N	R M1	14	80	N	2	2	4
20	61	M	3	1	Y	L M2	4	98	Y (Penum, iaTPA)	2	2	0
21	70	M	24	18	N	L M1	6	70	N	1	3	4
22	73	M	9	16	N	L M2	9	80	Y (Penumbra)	4	3	6
23	26	F	3	0	Y	R ICA	9	30	N	1	3	0
24	77	F	8	4	Y	R M1	7	26	N	1	3	1
25	65	M	15	11	Y	R M2	6.5	32	N	1	2	2
26	45	F	23	5	N	L M1	8	55	N	1	3	1
27	57	M	20	20	Y	R M2	6.5	80	Y (TREVO, iaTPA, Penum)	2	2	NA
28	54	M	12	0	N	L M1	8.5	65	N	5	3	0
29	56	F	17	5	Y	L M1	6	80	N	3	2	2
30	65	M	26	17	N	L ICA	7.5	75	Y(Merci)	3	3	NA
31	71	F	5	5	N	L M1	13.5	25	N	1	3	1

*Rescue treatment: procedures used in case the recanalization failed with attempt of one thrombectomy system. These include intra-arterial thrombolysis, angioplasty, and other thrombectomy systems than the initially attempted one.

**Table 2:** Recanalization grade as compared with NIHSS outcome at time of discharge.

	Good	Fair	Poor
TIMI 3	**15**	2	4
TIMI 2	3	*4 *	*1 *
TIMI 0 or 1	0	*0 *	*2 *

Bold emphasizes 15 of 18 patients (83%) with good NIHSS outcome at discharge had TIMI 3, while 7 out of 13 patients (54%) with fair or poor outcome did not achieve complete recanalization (TIMI 2 or 1).

Table graphically shows that TIMI 3 perfusion was a statistically significant predictor of good outcome at discharge (*P* = 0.012).

**Table 3:** Baseline demographic and clinical characteristics of the patients.

	Solitaire (*n* = 31)	Merci/Penumbra (*n* = 20)	
Age (years; range)	64.1 (32–87)	67.5 (31–85)	*P* = 0.02
Sex (% male)	74%	65%	*P* = 0.07
NIHSS score (Mean; Range)	13.7 (3–26)	14.3 (8–22)	*P* = 0.17
Body-mass index (mean)	28	29	*P* = 0.03
ivTPA administration	42%	45%	*P* = 0.02
Medical history			
HTN	75%	70%	
DM	17%	25%	
Smoking	42%	40%	
Atrial Fibrillation	35%	45%	
Use of antiplatelet or anticoagulation	48%	55%	
Most proximal occlusion location			
Internal carotid artery	13%	25%	
M1 middle cerebral artery	51%	50%	
M2 middle cerebral artery	29%	20%	
Posterior circulation	6%	5%	
Occlusion side (left)	61%	55%	
Time to arterial puncture (min; range)	432 min (242–962 min)	351 min (240–600 min)	

**Table 4:** Results of Solitaire tabulated for area of salvage, recanalization rate, intervention time and clinical results.

Patient	Age	Gender	NIHSS on arrival	NIHSS at 24 h	ivTPA	Location of thrombus	% Salvage	Time to reperfusion (hrs)	Procedure time (min)	TIMI	Symptomatic ICH	mRS
1	87	F	18	13	N	R M2	61	17	42	3	N	4
2	64	M	12	7	N	L M1	90.7	11	90	3	N	1
3	73	M	12	8	Y	L M1	1	7	36	3	N	2
4	32	M	18	5	Y	R M2	83.5	5	33	3	N	0
5	50	M	21	7	N	R ICA	61.4	5	65	2	N	1
6	52	M	7	3	N	Basilar	NA	6	73	3	N	0
7	76	M	12	6	N	R M1	95.2	10	44	3	N	2
8	77	M	13	3	Y	R M1	1	6	65	2	N	0
9	62	M	9	7	N	L M2	1	5	70	2	N	2
10	77	F	13	6	Y	L ICA	96.9	10	80	3	N	2
11	48	M	12	1	N	R M2	59	6	20	3	N	0
12	82	M	9	4	Y	L M1	82.1	5	26	3	N	4
13	69	M	9	9	N	L vert	NA	6	65	1	N	4
14	63	F	21	16	N	L M1	73.9	5	41	3	N	1
15	87	M	19	27	Y	R M1	57.6	5	54	0	N	6
16	63	M	18	10	N	R M1	NA	11	52	3	N	4
17	71	M	13	7	Y	R ICA	95.7	7.5	57	2	N	0
18	74	M	10	10	N	R M1	1	16	105	3	N	6
19	61	M	15	13	N	R M1	86.6	14	80	2	N	4
20	61	M	3	2	Y	L M2	90.9	4	98	2	N	0
21	70	M	24	18	N	L M1	91.5	6	70	3	N	4
22	73	M	9	14	N	L M2	NA	9	80	3	N	6
23	26	F	3	0	Y	R ICA	88.8	9	30	3	N	0
24	77	F	8	5	Y	R M1	81.1	7	26	3	N	1
25	65	M	15	11	Y	R M2	90.6	6.5	32	2	N	2
26	45	F	23	17	N	L M1	82.8	8	55	3	N	1
27	57	M	20	20	Y	R M2	39.3	6.5	80	2	N	NA
28	54	M	12	5	N	L M1	97.8	8.5	65	3	N	0
29	56	F	17	18	Y	L M1	28.9	6	80	2	N	2
30	65	M	26	26	N	L ICA	NA	7.5	75	3	N	NA
31	71	F	5	7	N	L M1	92.9	13.5	25	3	N	1

Average							81.9					

**Table 5:** Data for 20 comparison group who received Merci/Penumbra treatment.

Patient	Age	Sex	IVtpA	NIH A	% Salvage	Location of clot	Time to intervention	Intervention time	TIMI	Symptomatic ICH	mRS at F/U
1	73	M	1	15	80.7	L M1	7	75	3	Y	2
2	85	F	0	14	73.4	L ICA	10	77	0	N	4
3	53	M	1	11	76.4	R M1	9	69	2	N	4
4	86	F	0	14	59.0	R ICA	8	88	1	N	6
5	47	M	0	14	52.4	R M2	NA	48	0	N	6
6	58	M	0	22	81.0	R M1	6	72	1	N	6
7	49	M	0	21	86.5	L M1	NA	28	3	N	3
8	86	F	1	16	83.2	L M1	4	33	3	N	2
9	63	M	1	12	97.2	R ICA	5	80	3	N	1
10	67	M	0	14	78.7	R M1	5	38	2	N	6
11	59	F	1	15	96.0	R M1	5	65	3	N	2
12	78	M	0	16	27.7	L M1	5	73	2	N	6
13	68	F	0	20	48.8	L ICA	NA	111	3	N	6
14	68	M	1	22	36.8	L M1	4	113	2	Y	6
15	59	F	1	16	58.6	R M1	4	57	1	N	6
16	69	M	1	20	67.5	R ICA	7	100	3	Y	3
17	75	M	0	12	92.6	R M1	4	59	3	N	2
18	59	F	1	12	87.9	R M1	4	85	2	N	6
19	62	M	1	10	92.5	R ICA	7.5	110	3	N	1
20	67	M	1	8	61.7	L M2	5	38	3	N	2

Average					**71.9**		5.85	**70.95**			

**Table 6:** The outcomes of the present study compared to the results from large mechanical thrombectomy trials.

Present study		Merci [9]	Multi-Merci [8]	Penumbra pivotal stroke [3]	SWIFT trial [10] (solitaire arm)	TREVO2 trial [7] (TREVO2 arm)
Sample, *n*	31	141	164	125	58	88
Recanalization, *n* (%)	29 (93.5%)*	68 (48.2%)	112 (68.2%)	102 (81.6%)	48 (88.9%)	76 (86%)
symptomatic ICH, *n* (%)	0 (0)	11 (7.8%)	16 (9.7%)	14 (11.2%)	1 (1.7%)	4 (4%)
3 month mortality, *n* (%)	3 (10.3%)	61 (43.2%)	56 (34.1%)	31 (32.8%)	10 (17.2%)	29 (33%)
3 month mRS ≤ 2, *n* (%)	20 (69%)	39 (27.6%)	59 (35.9% )	52 (41.6%)	32 (58.2%)	38/85 (40%)

*This is the final recanalization rate which includes rescue treatment with other devices as well as iaTPA. With Solitaire FR device alone, the recanalization rate was 79.5%.
